# Development and validation of a prediction model for insulin-associated hypoglycemia in non-critically ill hospitalized adults

**DOI:** 10.1136/bmjdrc-2017-000499

**Published:** 2018-03-02

**Authors:** Nestoras Nicolas Mathioudakis, Estelle Everett, Shuvodra Routh, Peter J Pronovost, Hsin-Chieh Yeh, Sherita Hill Golden, Suchi Saria

**Affiliations:** 1 Division of Endocrinology, Diabetes and Metabolism, School of Medicine, Johns Hopkins University, Baltimore, Maryland, USA; 2 Department of Anesthesiology and Critical Care Medicine, School of Medicine, Johns Hopkins University, Baltimore, Maryland, USA; 3 Department of Epidemiology, Bloomberg School of Public Health, Johns Hopkins University, Baltimore, Maryland, USA; 4 Department of Computer Science, Whiting School of Engineering, Johns Hopkins University, Baltimore, Maryland, USA

**Keywords:** hypoglycemia, hospital management, prediction, insulin

## Abstract

**Objective:**

To develop and validate a multivariable prediction model for insulin-associated hypoglycemia in non-critically ill hospitalized adults.

**Research design and methods:**

We collected pharmacologic, demographic, laboratory, and diagnostic data from 128 657 inpatient days in which at least 1 unit of subcutaneous insulin was administered in the absence of intravenous insulin, total parenteral nutrition, or insulin pump use (index days). These data were used to develop multivariable prediction models for biochemical and clinically significant hypoglycemia (blood glucose (BG) of ≤70 mg/dL and <54 mg/dL, respectively) occurring within 24 hours of the index day. Split-sample internal validation was performed, with 70% and 30% of index days used for model development and validation, respectively.

**Results:**

Using predictors of age, weight, admitting service, insulin doses, mean BG, nadir BG, BG coefficient of variation (CV_BG_), diet status, type 1 diabetes, type 2 diabetes, acute kidney injury, chronic kidney disease (CKD), liver disease, and digestive disease, our model achieved a c-statistic of 0.77 (95% CI 0.75 to 0.78), positive likelihood ratio (+LR) of 3.5 (95% CI 3.4 to 3.6) and negative likelihood ratio (−LR) of 0.32 (95% CI 0.30 to 0.35) for prediction of biochemical hypoglycemia. Using predictors of sex, weight, insulin doses, mean BG, nadir BG, CV_BG_, diet status, type 1 diabetes, type 2 diabetes, CKD stage, and steroid use, our model achieved a c-statistic of 0.80 (95% CI 0.78 to 0.82), +LR of 3.8 (95% CI 3.7 to 4.0) and −LR of 0.2 (95% CI 0.2 to 0.3) for prediction of clinically significant hypoglycemia.

**Conclusions:**

Hospitalized patients at risk of insulin-associated hypoglycemia can be identified using validated prediction models, which may support the development of real-time preventive interventions.

**Significance of this study**

**What is already known about this subject?**

- Inpatient hypoglycemia can be predicted with only a modest degree of accuracy on the basis of body weight, renal function, and hospital insulin doses.

**What are the new findings?**

- Using a broad number of covariates, including age, weight, admitting service, insulin doses, blood glucose data, diabetes type, renal and liver function, and other admission diagnoses, insulin-associated hypoglycemia can be predicted within a 24-hour time window with a good degree of accuracy.

**How might these results change the focus of research or clinical practice?**

- Development of a real-time informatics alert from an accurate prediction model could be used clinically to prevent insulin-associated hypoglycemia, a potentially serious complication among hospitalized patients.

Hypoglycemia is a common occurrence in hospitalized patients and is linked to multiple adverse clinical outcomes and mortality.^1^ Acute hypoglycemia can provoke cardiac ischemia and arrhythmias, as well as neurologic harm ranging in severity from altered cognition or irritability to focal neurologic deficits, loss of consciousness, stroke, seizures, and coma.^2^ Besides these potentially life-threatening complications, hypoglycemia can be a source of patient dissatisfaction and worry.^1^ Approximately half of hypoglycemic events in the hospital are iatrogenic, usually resulting from insulin treatment.^3^ In observational studies of hospitalized patients, both spontaneous and iatrogenic hypoglycemia are associated with increased mortality.^3–6^ Considering that 20%–40% of hospitalized patients require glucose-lowering medications,^1^ prevention of iatrogenic hypoglycemia is a significant patient safety issue and a major challenge to our healthcare system.

Insulin is the recommended therapy for glycemic management in the non-critical care setting, with discontinuation of non-insulin antihyperglycemic agents encouraged for the majority of patients.^7^ In contrast to the intensive care unit (ICU), where insulin adjustments may be driven by nurse-managed protocols, insulin titration in the non-ICU setting is prescriber-driven and requires evaluation of a complex set of clinical, laboratory, and pharmacologic parameters. Unfortunately, therapeutic inertia—failure to reduce or modify insulin therapy in patients with downward trending blood glucose (BG) readings—is a common cause of insulin-associated hypoglycemia. In over 60% of severe hypoglycemic events, antecedent mild hypoglycemia was observed without any change in diabetes medications.^8^ Even more concerning, clinicians often fail to modify insulin doses in patients who experience overt hypoglycemia. A retrospective study found that only 44% of patients had the recommended 20% reduction in the insulin total daily dose following a hypoglycemic event.^9^

The purpose of this study was to develop and validate a prediction model for insulin-associated hypoglycemia in non-critically ill hospitalized adults. A previous logistic regression model developed using data from 3028 inpatients achieved 54% sensitivity at detecting hypoglycemia using a BG cut-off of 60 mg/dL, missing 46% of acute hypoglycemic events.^10^ By using a much larger data set and more predictor variables, we hoped to achieve a model with greater predictive accuracy, moving closer to the goal of a real-time alerting or reporting system integrated into the electronic medical record (EMR) to identify inpatients at high risk of incident insulin-associated hypoglycemia in a clinically relevant time window that would permit prophylactic changes to the insulin regimen.

## Research design and methods

### Data source

This was a cross-sectional study conducted at Johns Hopkins Hospital, a 1300-bed tertiary care academic medical center in Baltimore, Maryland. Using data from our prior EMR, Sunrise POE, we identified hospitalized adults in the non-critical care, non-obstetrical setting with an admission date on or after 1 January 2013 and a discharge date on or before 31 December 2015. Admissions missing a date of discharge or patient weight measurements were excluded. The primary exposure of interest was treatment with subcutaneous insulin, defined as any administered long-acting, intermediate-acting, rapid-acting, or premixed insulin. At our institution, the formulary long-acting insulin is glargine, the intermediate-acting insulin is Neutral Protamine Hagedorn (NPH), the rapid-acting insulin is aspart, and the premixed insulin is NPH/regular 70/30. Admissions in which subcutaneous insulin was not administered at any time during hospitalization were excluded.

For eligible admissions, we defined patient-days as 24-hour intervals relative to the admission date and time (online supplementary figure S1). We defined insulin-treated patient-days as patient-days in which at least 1 unit of subcutaneous insulin was administered. We excluded those insulin-treated patient-days in which there was concurrent use of intravenous insulin (as these patients are already under close observation and managed via protocol with hourly BG checks), total parenteral nutrition (TPN; as intravenous insulin is often an additive in the parenteral nutrition), insulin pump use (as we were unable to easily capture patient-administered insulin pump doses from our EMR), or if the insulin-treated patient-day was the date of discharge (as no outcome data would be available). All remaining insulin-treated patient-days were considered index days used for prediction of hypoglycemic outcomes on the next patient-day. Although non-index days were excluded as a unit of observation for prediction, they were included as observations for outcome ascertainment if the previous day was an index day.

10.1136/bmjdrc-2017-000499.supp1 Supplementary file 1

### Outcomes

The primary outcomes were biochemical or clinically significant hypoglycemia, defined as at least one serum or fingerstick BG of ≤70 mg/dL and <54 mg/dL occurring in a prediction horizon of 24 hours after an index day, respectively.^11^ Since the degree of hypoglycemia might be influenced by different clinical predictors, we developed separate prediction models for each hypoglycemic outcome.

### Predictors

Candidate predictors of hypoglycemia were selected based on clinical knowledge and previous studies, and with consideration of ease of data extraction from our EMR. Online supplementary table S1 summarizes the definitions, data sources, and timing of collection for each of the predictor variables. Demographic predictors included age, sex, race, and admitting service (medical vs surgical). At our institution, consistent with current practice guidelines, it is recommended that non-insulin antihyperglycemic medications be discontinued and insulin therapy initiated for patients with hyperglycemia persisting for 24–48 hours.^7^ Therefore, with the exception of subcutaneous insulin, we did not collect information about other hospital-administered antihyperglycemic medications. Administered subcutaneous insulin was categorized as basal, nutritional, and correctional. Although insulin doses were normalized per body weight (unit/kg/index day) in our prediction models, weight was included separately as an independent variable.

For most insulin-treated hospitalized patients on medical/surgical wards, BG measurements are typically obtained four times daily (before each meal and at bedtime) or every 4 hours if nil per os (NPO). We evaluated several glycemic measures as predictors of incident hypoglycemia. Mean BG and coefficient of variation of BG (CV_BG_) and nadir BG were summarized both on the index day and for all inpatient days up to and including the index day. Both index day and admission-level glycemic measures were evaluated as predictors.

Using relevant *International Classification of Disease*s (ICD-9) diagnostic codes, we generated categories of clinical conditions that could affect glucose regulation. We categorized type 1 diabetes mellitus and postsurgical hypoinsulinemia as insulin-deficient states. Without information about home insulin use, we could not identify patients with type 2 diabetes who were insulin-deficient. Acute kidney injury (AKI) and chronic kidney disease (CKD) predispose to hypoglycemia via reduced insulin clearance and reduced gluconeogenesis.^12^ Liver failure causes hyperglycemia due to increased insulin resistance^13^ and in decompensated failure may cause hypoglycemia due to reduced gluconeogenesis.^14 15^ Chronic alcohol use can cause hypoglycemia due to depletion of glycogen stores from prolonged fasting and inhibition of gluconeogenesis.^16^ We combined alcohol dependency and end-stage liver disease into a category of liver disease. Congestive heart failure is another less common hypoglycemic risk factor.^17–19^ Acute pancreatitis and pancreatic cancer have both been associated with hyperglycemia (ie, pancreatogenous diabetes).^20^ Given the low prevalence of each of these conditions, we combined them into one category. Steroids, which are commonly used in hospitalized patients, contribute to hyperglycemia via insulin resistance.^21^ We evaluated systemic steroid use as a binary predictor, but did not have information about steroid dose to evaluate the effect of steroid tapers on insulin requirements and hypoglycemia risk. Given the very low ICD coding for sepsis among diagnoses (0.07% of index days), sepsis was not included as a predictor in this non-critically ill population. Adrenal insufficiency is another known hypoglycemic risk factor; however, coding for this condition was virtually absent.

Reduced carbohydrate intake following insulin administration is a common cause of hypoglycemia. In addition to diet orders reflecting varying degrees of carbohydrate intake, we created a category of digestive diseases to include several conditions (nausea, vomiting, abdominal pain, intestinal obstruction) that could influence carbohydrate intake. Although we considered using diagnostic codes associated with malnourishment (eg, failure to thrive, cachexia), coding for these conditions was exceedingly low.

### Missing data

After excluding admissions lacking discharge date and weight information, we had nearly complete information about the predictors and outcomes for our analysis. The variables missing information were admission diagnosis codes and renal laboratory data on patient day 1, which were missing from 10.1% and 30.3% of records, respectively. Patient-days missing admission diagnoses were included in the analysis; however, the diagnoses used to classify clinical conditions as predictor variables were assumed to be absent for these records. Similarly, we assumed that most patients with CKD would have had laboratory assessment of renal function obtained within the first 24 hours of admission. If a glomerular filtration rate measurement was missing, the patient-day was included in the analysis, but the patient was classified as not having CKD.

### Statistical analysis and methods

We adhered to the Transparent Reporting of a multivariable prediction model for Individual Prognosis Or Diagnosis guidelines in the design of this study and reporting of results.^22^ We used split-sample internal validation with 70% and 30% of the index days used for model development and validation, respectively. Records were sorted chronologically by discharge date, and the discharge date corresponding approximately to the 70th percentile of records was used to split the cohort such that it did not partition an individual patient admission. This approach was selected because it allows for non-random variation that would be encountered in the real-world clinical setting if such a model were to be used prospectively for real-time event prediction.

A detailed description of our model building strategy is provided in the online supplementary materials. In brief, we used a combination of various selection processes, including trial and error, automated stepwise selection, and evaluation of the information criteria to develop two multivariable logistic regression models: model 1 for prediction of a BG ≤70 mg/dL (biochemical hypoglycemia) and model 2 for a BG <54 mg/dL (clinically significant hypoglycemia). Using the multivariable regression equations for the final models, the probability of hypoglycemia was calculated in the development data set. An empirical probability cut-point was selected using Youden’s Index, which maximizes the sum of sensitivity and specificity.^23^ The probability of hypoglycemia was then calculated in the validation cohort using the coefficients from the regression equation derived from the development data set. The empirical probability cut-points were then applied to the validation data set, such that a predicted probability at or above the probability cut-point was used to classify the index day as ‘at risk’ for hypoglycemic event.

Model performance was assessed by comparing true disease status versus classified risk in the validation set. Discrimination was assessed using the c-statistic, which is equal to the area under the receiver operating characteristic curve. c-Statistic values >0.7, >0.8, and >0.9 are considered acceptable, excellent, and outstanding discrimination, respectively.^24^ Positive and negative predictive values, which are dependent on disease prevalence, indicate the probability of a positive/negative test result among those with/without the disease, respectively.^25^ Positive and negative likelihood ratios are test characteristics that are independent of disease prevalence and provide information about whether a test result changes the probability of the outcome.^25^ Positive likelihood ratios of >2, >5, and >10 indicate slight, moderate, and large increases in the likelihood of the outcome with a positive result, respectively; conversely, negative likelihood ratios of <0.5, <0.2, and <0.1 indicate slight, moderate, and large decreases in the likelihood of the outcome with a negative result.^26^

Statistical analyses were performed using Stata Statistical Software V.14.2. P<0.05 was considered statistically significant.

## Results

### Characteristics of study population

**Figure 1:**
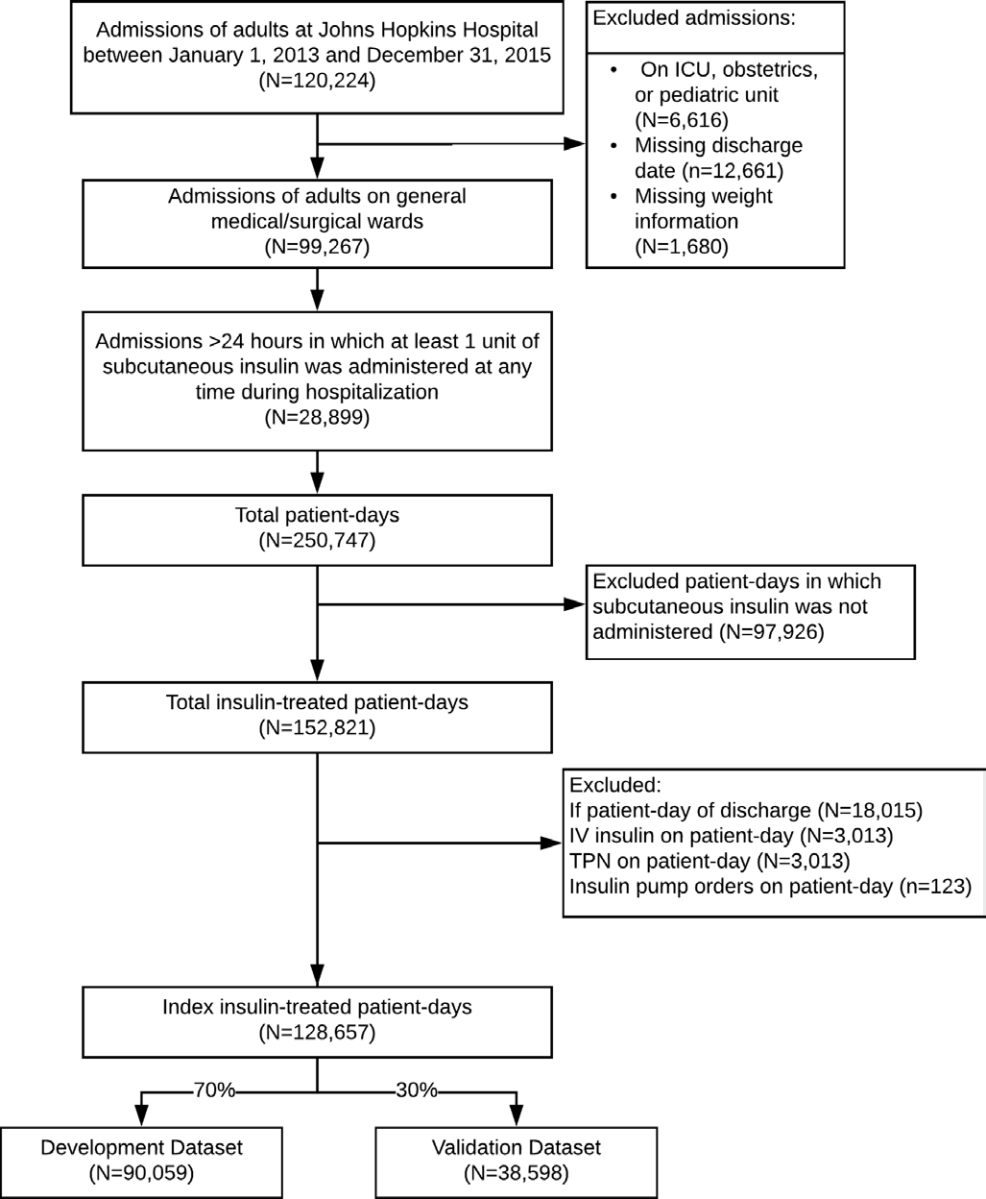
Study flow chart. ICU, intensive care unit; TPN, total parenteral nutrition.

Among 120 224 patient admissions during the study period, 28 899 (24%) had administration of at least 1 unit of subcutaneous insulin (figure 1). Of these eligible admissions, there were a total of 250 747 patient-days, of which 152 821 (61%) were insulin-treated patient-days. After excluding discharge days and days with intravenous insulin, TPN, or insulin pump orders, 128 657 (84%) of the insulin-treated days were identified as index days for prediction of a hypoglycemic outcome within the next 24 hours.

The baseline characteristics and hypoglycemic outcomes of the study cohort are shown in table 1. The prevalence of biochemical and clinically significant hypoglycemia was 4.2% and 1.2% of patient-days, respectively. The median length of stay was 6 days, with a slightly higher admission rate to surgical compared with medical services. The median age was 61.2 years, with a slight male predominance. The majority of patients were white (53.8%), followed by black (36.2%), other races (7.5%), and Asian (2.5%). Type 2 diabetes and insulin-deficient diabetes (type 1/pancreatectomy) were present in 56.4% and 4.2% of admissions, respectively.

**Table 1:** Baseline characteristics and hypoglycemic outcomes of study population

Variable	Results reported per		Development data set	Validation data set	Full cohort
	Admission	Index day			
Insulin-treated patient-days, n		x	90 059	38 598	128 657
Patient admissions, n	x		18 867	7399	26 266
Unique patients, n	x		13 360	5902	18 196
Hypoglycemic outcome, n (%)					
Any BG ≤70 mg/dL		x	3935 (4.4)	1517 (3.9)	5452 (4.2)
Any BG <54 mg/dL		x	1081 (1.2)	415 (1.1)	1496 (1.2)
Nadir BG, mg/dL		x	61 (52–66)	61 (53–66)	61 (52–66)
LOS, days	x		6 (3–10)	6 (4–11)	6 (3–11)
Admitting service, n (%)	x				
Medicine	x		9115 (48.3)	3324 (44.9)	12 439 (47.4)
Surgery	x		9752 (51.7)	4075 (55.1)	13 827 (52.6)
Age, years	x		61.1 (51.3–70.2)	61.5 (52.2–69.8)	61.2 (51.6–70.1)
Sex, male/female (%)	x		52.5/47.5	54.1/45.9	52.9/47.1
Race, n (%)	x				
White	x		10 125 (53.7)	3997 (54.0)	14 122 (53.8)
Black	x		6923 (36.7)	2589 (35.0)	9512 (36.2)
Asian	x		428 (2.3)	224 (3.0)	652 (2.5)
Other	x		1391 (7.4)	589 (8.0)	1980 (7.5)
Admission weight, kg	x		81.6 (67.8–98.1)	82.2 (68.2–98.0)	81.7 (68.0–98.1)
Insulin doses, unit/kg/day					
Total daily dose		x	0.10 (0.03–0.32)	0.11 (0.03–0.32)	0.10 (0.03–0.32)
Basal		x	0.00 (0.00–0.15)	0.00 (0.00–0.16)	0.00 (0.00–0.16)
Nutritional		x	0.00 (0.00–0.00)	0.00 (0.00–0.00)	0.00 (0.00–0.00)
Correctional		x	0.05 (0.02–0.11)	0.05 (0.02–0.11)	0.05 (0.02–0.11)
High-dose SSI, n (%)		x	53 488 (59.4)	23 406 (60.6)	76 894 (59.8)
Diet orders, n (%)		x			
Nil per os		x	20 362 (22.6)	8765 (22.7)	29 127 (22.6)
Carbohydrate-controlled		x	8832 (9.8)	3450 (8.9)	12 282 (9.6)
Regular or other diet		x	58 198 (64.6)	25 034 (64.9)	82 232 (64.7)
Bolus tube feeds		x	2667 (3.0)	1349 (3.5)	4016 (3.1)
Steroid use, n (%)		x	39 451 (43.7)	17 809 (46.0)	57 260 (44.4)
Type 1 diabetes/pancreatectomy, n (%)	x		900 (4.8)	217 (2.9)	1117 (4.3)
Type 2 diabetes, n (%)	x		11 357 (60.2)	3434 (46.4)	14 791 (56.3)
Acute kidney injury, n (%)	x		191 (1.0)	41 (0.6)	232 (0.9)
Chronic kidney disease, n (%)	x				
Stage 3	x		3502 (18.6)	421 (5.7)	3921 (14.9)
Stage 4	x		1287 (6.8)	149 (2.0)	1450 (5.5)
Stage 5	x		919 (4.9)	89 (1.2)	1016 (3.9)
Liver failure, n (%)	x		153 (0.8)	58 (0.8)	211 (0.8)
Congestive heart failure, n (%)	x		431 (2.3)	93 (1.3)	524 (2.0)
Digestive disease, n (%)	x		935 (5.0)	294 (4.0)	1229 (4.7)
Pancreatic disease, n (%)	x		468 (2.5)	144 (2.0)	612 (2.3)
Index day glycemic measures					
Nadir BG, mg/dL		x	121 (100–147)	122 (101–148)	121 (100–147)
Mean BG, mg/dL		x	158 (134–194)	160 (136–197)	158 (135–195)
CV of BG, %		x	19.2 (12.8–27.8)	18.9 (12.8–27.1)	19.1 (12.8–27.6)
Number of BG measurements		x	5 (4–7)	6 (5–7)	5 (5–7)
Admission glycemic measures*					
Nadir BG, mg/dL	x		91 (73–115)	91 (73–113)	91 (73–114)
Mean BG, mg/dL	x		161 (139–194)	163 (141–196)	162 (139–195)
CV of BG, %	x		24.8 (19.0–32.5)	25.2 (19.3–32.2)	25.0 (19.1–32.4)

*Summarized for all patient-days prior to and including index day. Data are median (interquartile range) and n (%).

BG, blood glucose; CV, coefficient of variation; LOS, length of stay; SSI, sliding scale insulin.

Overall, administered insulin doses were relatively low, with median (IQR) total daily dose (TDD) of 0.10 (0.03–0.32) units/kg. The majority of subcutaneous insulin was provided in the form of basal and correctional insulin. Despite hospital protocols and computerized ordersets encouraging use of a basal-bolus insulin regimen, use of nutritional insulin was exceedingly low. For example, the 90th percentile of nutritional insulin dose was only 0.17 units/kg/day. High-dose sliding scale insulin (SSI) was used in 59.8% of index days. There was a high prevalence of steroid use on the index day (44.4%).

Regarding conditions associated with hypoglycemia, CKD was most prevalent, with 14.9%, 5.5%, and 3.9% having stage 3, stage 4, and stage 5 CKD, respectively. AKI was an admission diagnosis in 0.9%. Digestive diseases affecting nutritional intake were present in 4.7%. Liver diseases and congestive heart failure (CHF) were admission diagnoses in 0.8% and 2.0%, respectively. NPO diet was ordered on 22.6% of index days.

With respect to glycemic measures, the mean and nadir BG on the index day were 158 mg/dL and 121 mg/dL, and the mean and nadir BG during admission and up to index day were 162 mg/dL and 91 mg/dL, respectively. As expected, glycemic variability (CV_BG_) was greater when summarized on all hospital days up to the index day compared with the index day alone (25.0% vs 19.1%). Overall, the development and validation data sets were very similar with respect to the clinical predictors, with the exception of diabetes and CKD, which were more prevalent in the development data set.

### Model parameters

Table 2 shows the fully adjusted models with ORs, coefficients, and intercepts. The univariate associations for each predictor are shown in online supplementary table S2. For biochemical hypoglycemia, there were a total of 3935 events and 44 variables in the model, with an event per variable (EPV) ratio of 89. For clinically significant hypoglycemia, there were a total of 1081 events and 35 variables, with an EPV ratio of 30.9. Thus, both models exceeded the recommended EPV of 10 or more.

**Table 2:** Logistic regression models with ORs and coefficients from validation data sets

	Unit of change	Spline knot	Model 1: BG≤70 mg/dL		Model 2: BG<54 mg/dL	
			OR (95% CI)	Coefficient	OR (95% CI)	Coefficient
Intercept				2.140		0.580
Age	10 years					
Age_1_		≤40	**1.07 (0.96 to 1.20)**	0.066	–	–
Age_2_		>40	**0.96 (0.92 to 0.99)**	−0.045	–	–
Female	–	–	–	–	**0.76 (0.66 to 0.86)**	−0.280
Weight	10 kg					
Weight_1_		≤80	**0.87 (0.84 to 0.91)**	−0.136	**0.85 (0.80 to 0.91)**	−0.160
Weight_2_		>80	**0.92 (0.90 to 0.95)**	−0.079	**0.86 (0.81 to 0.91)**	−0.151
Admission to surgical service			**0.91 (0.85 to 0.98)**	−0.091	–	–
Basal insulin dose	0.1 units/kg					
Basal_1_		≤0.2	**1.85 (1.75 to 1.97)**	0.617	**2.01 (1.79 to 2.25)**	0.697
Basal_2_		≤0.8	**1.14 (1.10 to 1.17)**	0.130	**1.15 (1.09 to 1.21)**	0.141
Basal_3_		≤1.3	0.95 (0.85 to 1.06)	−0.050	0.95 (0.80 to 1.14)	−0.046
Basal_4_		≤1.6	1.29 (0.92 to 1.78)	0.249	1.84 (1.15 to 2.95)	0.609
Basal_5_		>1.6	0.95 (0.71 to 1.28)	0.755	0.29 (0.07 to 1.28)	−1.238
Nutritional insulin dose	0.1 units/kg					
Nutritional_1_		≤0.6	**1.05 (1.02 to 1.08)**	0.048	1.05 (1.00 to 1.10)	0.049
Nutritional_2_		≤0.9	1.05 (0.90 to 1.23)	0.048	1.04 (0.80 to 1.33)	0.035
Nutritional_3_		≤1.1	**0.59 (0.36 to 0.94)**	−0.534	0.51 (0.22 to 1.17)	−0.674
Nutritional_4_		>1.1	1.07 (0.86 to 1.35)	0.071	1.19 (0.86 to 1.65)	0.176
Correctional insulin dose	0.1 units/kg					
Correctional_1_		≤0.04	**0.69 (0.49 to 0.97)**	−0.365	–	–
Correctional_2_		≤0.7	1.04 (0.99 to 1.10)	0.041	–	–
Correctional_3_		≤0.9	0.71 (0.39 to 1.30)	−0.336	–	–
Correctional_4_		>0.9	1.17 (0.79 to 1.72)	0.159	–	–
High-dose SSI			**1.14 (1.03 to 1.27)**	0.135	**1.18 (0.97 to 1.45)**	0.169
Index day mean BG	10 mg/dL					
Mean_1_		≤100	**0.73 (0.64 to 0.83)**	−0.312	**0.80 (0.66 to 0.97)**	−0.220
Mean_2_		≤150	**0.86 (0.83 to 0.89)**	−0.155	**0.89 (0.84 to 0.94)**	−0.122
Mean_3_		>150	0.99 (0.97 to 1.01)	−0.011	1.03 (1.01 to 1.05)	0.030
Index day nadir BG	10 mg/dL					
Nadir_1_		≤88	0.99 (0.95 to 1.03)	−0.011	0.91 (0.85 to 0.97)	−0.099
Nadir_2_		≤100	**0.64 (0.58 to 0.71)**	−0.440	**0.80 (0.66 to 0.96)**	−0.228
Nadir_3_		>100	**0.93 (0.91 to 0.95)**	−0.070	**0.93 (0.89 to 0.96)**	−0.076
Index day CV of BG	10%					
CV_1_		≤10	0.93 (0.61 to 1.42)	−0.077	1.04 (0.44 to 2.47)	0.035
CV_2_		≤20	**1.72 (1.46 to 2.02)**	0.541	**1.51 (1.10 to 2.09)**	0.417
CV_3_		>20	1.01 (0.96 to 1.05)	0.006	1.02 (0.94 to 1.10)	0.017
Admission nadir BG	10 mg/dL					
Admission nadir_1_		≤100	**0.87 (0.86 to 0.89)**	−0.134	**0.86 (0.83 to 0.90)**	−0.147
Admission nadir_2_		≤400	1.03 (1.01 to 1.05)	0.029	1.04 (1.00 to 1.07)	0.035
Admission nadir_3_		>400	0.84 (0.58 to 1.19)	−0.180	0.88 (0.63 to 1.24)	−0.122
Admission CV of BG	10%					
Admission cv_1_		≤18	**0.78 (0.63 to 0.96)**	−0.249	**0.73 (0.49 to 1.09)**	−0.311
Admission cv_2_		>18	**1.19 (1.14 to 1.24)**	0.171	**1.17 (1.10 to 1.25)**	0.159
Diet orders						
NPO			1.00 (ref)	–	1.00 (ref)	–
Carbohydrate-controlled			**0.80 (0.71 to 0.92)**	−0.218	**0.67 (0.53 to 0.84)**	−0.406
Regular or other			**0.79 (0.72 to 0.86)**	−0.235	**0.69 (0.59 to 0.81)**	−0.373
Bolus tube feeds			**0.68 (0.54 to 0.86)**	−0.382	**0.76 (0.51 to 1.15)**	−0.268
Type 1 diabetes/pancreatectomy			**1.43 (1.28 to 1.59)**	0.357	**2.04 (1.73 to 2.39)**	0.712
Type 2 diabetes			**1.25 (1.13 to 1.38)**	0.224	**1.29 (1.07 to 1.55)**	0.253
AKI			1.27 (0.99 to 1.63)	0.238	–	–
CKD						
None			1.00 (ref)	–	1.00 (ref)	–
Stage 3			**1.25 (1.15 to 1.37)**	0.224	**1.37 (1.16 to 1.61)**	0.314
Stage 4			**1.52 (1.37 to 1.69)**	0.418	**1.80 (1.49 to 2.17)**	0.588
Stage 5			**1.76 (1.56 to 1.99)**	0.565	**2.21 (1.80 to 2.73)**	0.795
Liver disease			**0.70 (0.48 to 1.01)**	−0.363	–	–
Digestive disease			1.20 (1.04 to 1.39)	0.184	–	–
Steroids on index day			**–**	–	**0.84 (0.74 to 0.96)**	−0.170

Bolded values signify P<0.05.

AKI, acute kidney; BG, blood glucose; CKD, chronic kidney disease; CV, coefficient of variation; NPO, nil per os; ref, reference; SSI, sliding scale insulin.

Predictors associated with reduced risk of hypoglycemia in one or both models included female sex, increasing age over 40 years, admission to surgical service, higher weight, higher index day mean and nadir BG, higher admission nadir BG, non-NPO diet, liver disease, and steroid use. Predictors associated with increased risk of hypoglycemia in one or both models included increasing basal insulin doses (in range of ≤0.8 units/kg), increasing nutritional insulin doses (in range of ≤0.6 units/kg), use of high-dose SSI, higher index day and admission CV_BG_, NPO diet, type 1 diabetes/pancreatectomy, type 2 diabetes, CKD, and digestive diseases.

There was significantly lower total insulin use in surgical compared with medical patients, with median (IQR) doses of 0.07 (0.02–0.24) and 0.14 (0.04–0.38) unit/kg/day, respectively (P<0.001). Lower insulin doses in surgical patients may have been related to higher prevalence of NPO status in this group: 54.8% of index days were NPO in surgery patients compared with 45.2% in medical patients (P<0.001).

Glycemic measures were the strongest predictors of hypoglycemia risk. For example, in model 1, for each 10 mg/dL increase in index day mean BG, the reductions in the adjusted odds of hypoglycemia were 27%, 14%, and 1% in the ranges of BG of ≤100 mg/dL, >100 and ≤150 mg/dL, and >150 mg/dL, respectively. Similarly, each 10% increase in the admission CV_BG_ beyond 18% was associated with a 19% increase in the adjusted odds of hypoglycemia.

Online supplementary tables S3 and S4 provide an explanation on how to use each model to calculate prediction for an individual patient-day using mock case examples.

### Model performance

Table 3 shows the performance characteristics for the prediction models. The selected probability cut-points for prediction of biochemical and clinically significant hypoglycemia were 0.038 and 0.009, respectively. At these cut-points, model 1 achieved a sensitivity of 74.6% and a specificity of 78.5%, with corresponding c-statistic of 0.77, indicating good performance. The positive and negative predictive values were 12.4% and 98.7%, respectively. The positive and negative likelihood ratios were 3.5 and 0.3, respectively, consistent with small to moderate effect in the likelihood of the outcome with a positive or negative test result. Model 2 performed slightly better with a sensitivity of 81.9%, specificity of 78.6%, c-statistic of 0.80, positive likelihood ratio of 3.8, and negative likelihood ratio of 0.2.

**Table 3:** Performance of prediction models

	Model 1: BG≤70 mg/dL	Model 2: BG<54 mg/dL
Probability cut-point	0.038	0.009
c-Statistic at probability cut-point	0.77 (0.75–0.78)	0.80 (0.78–0.82)
Sensitivity (%)	74.6 (72.3–76.7)	81.9 (77.9–85.5)
Specificity (%)	78.5 (78.1–78.9)	78.6 (78.2–79.0)
Positive predictive value (%)	12.4 (11.7–13.1)	4.0 (3.6–4.4)
Negative predictive value (%)	98.7 (98.6–98.8)	99.8 (99.7–99.8)
Positive likelihood ratio	3.5 (3.4–3.6)	3.8 (3.7–4.0)
Negative likelihood ratio	0.3 (0.3–0.4)	0.2 (0.2–0.3)

BG, blood glucose.

## Conclusions

Using EMR data from a large patient population, we developed a model to predict biochemical and clinically significant hypoglycemia in hospitalized patients treated with subcutaneous insulin. Internal validation of our models revealed good performance for detection of hypoglycemic outcomes, with slightly greater accuracy in detection of clinically significant compared with biochemical hypoglycemia. Since all of the predictor variables are readily available in the EMR, our models could be used to develop a real-time informatics alert to prevent insulin-associated hypoglycemia in hospitalized patients, a potentially serious clinical outcome.

Among candidate predictors, we found that glycemic summary measures were the strongest predictors. Index day mean, nadir, and CV_BG_ were strongly associated with odds of hypoglycemia, as were the admission nadir BG and variability. Basal insulin doses were modestly associated with increased risk, but only for doses ≤0.8 units/kg/day. We suspect that patients requiring basal doses beyond this had some underlying cause of severe insulin resistance, such as morbid obesity or high-dose glucocorticoid use, which may have provided protection against hypoglycemia. Interestingly, nutritional insulin doses were only weakly associated with increased risk in the range <0.6 unit/kg/day, possibly owing to the overall low use of nutritional insulin in this cohort and lack of adequate power to detect effect sizes at larger doses. While use of high-dose SSI was associated with increased risk, administered correctional insulin doses were not.

A previous study found that body weight, creatinine clearance, basal insulin dose, basal-only dosing (without mealtime insulin), use of 70/30 insulin, and use of oral antidiabetic agents were predictors of hypoglycemia in hospitalized patients.^10^ Many of these predictors were also significant in our models; other predictor variables that we identified were type 1 diabetes/pancreatectomy, type 2 diabetes, liver disease, digestive conditions affecting nutritional intake, nutritional status, age, and admitting service. Not surprisingly, one variable that has been shown to be a strong determinant for hypoglycemia is a prior episode of hypoglycemia.^27 28^ In our models, prior episodes of hypoglycemia were captured in the admission nadir BG variable, which was indeed a strong predictor of hypoglycemia. In the BG range of ≤88 mg/dL, each 10 mg/dL decrease in the admission nadir BG was associated with a 13% and a 14% increase in the odds of biochemical and clinically significant hypoglycemia, respectively (table 2, admission nadir_1_).

In general, prediction in data sets where the prevalence of the target outcome is low (like insulin-associated hypoglycemia) tends to be challenging. A previously published model achieved a sensitivity of 61%, specificity of 65%, positive predictive value of 13% and negative predictive value of 95% for detection of a BG <70 mg/dL in hospitalized patients.^10^ Accordingly, the positive likelihood ratio was 1.7 and the negative likelihood ratio was 0.6. While the positive predictive value of that model (13%) was comparable with ours (12.4%), our positive likelihood ratio (3.5) was double. Unlike predictive value tests, the likelihood ratio is a test characteristic that does not depend on the prevalence of the disease in the population.^26^ Our models achieved positive likelihood ratios of 3.5 and 3.8, which would correspond to approximately 23% and 24% increases in the probability of biochemical and clinically significant hypoglycemia, respectively.^26^

Predictive models using hospital information systems have been shown to improve both the quality and cost of care in situations where patient conditions can change rapidly, such as detection of sepsis or septic shock, immediate cardiac arrest, ventilator-induced lung injury, and AKI.^29–31^ Based on their predictive model, Kilpatrick *et al* developed a real-time alert process to reduce rates of inpatient hypoglycemia. Their alert, augmented by nurse–physician collaboration, reduced rates of severe hypoglycemia by 68% in high-risk patients.^32^ The main limitations of alert systems are false alarms and alert fatigue, where providers become desensitized or even confused by alerts. Identifying the appropriate sensitivity and specificity of informatics alerts is an area of ongoing research.^33^

Although our models performed well, there were some limitations in our study, which if addressed in future studies could further enhance their predictive capability and clinical utility as a real-time alerting tool. Importantly, by using a time frame of 24 hours for our prediction window and aggregating information about insulin doses and glucose measures, we were unable to account for the duration of action of insulin on individual BG readings. For patients receiving only rapid-acting insulin (aspart), which has a duration of action of 4–6 hours, it is possible that spontaneous hypoglycemic episodes were misclassified as insulin-associated hypoglycemic events since they may have occurred outside the insulin’s duration of action but within the 24-hour prediction window. We were, unfortunately, unable to narrow down the prediction window to intervals less than 24 hours because the data set only included aggregate insulin information summarized by patient-day, rather than information about individual insulin doses. We are currently working on a new data set derived from our present EMR, EpicCare, to calculate the insulin dose on board relative to each BG reading, which will allow us to more accurately classify insulin-associated hypoglycemic events within more narrow prediction windows based on the pharmacologic actions of the different insulin types. Similarly, information about steroid doses would be useful, since hypoglycemia risk may be increased during steroid tapers. As this was a real-time prediction model, severity of illness and mortality indices (which are calculated after discharge) could not be used but would be expected to be important predictors. Although we did not have information about vital signs in our data set, we are considering using them as surrogate markers of illness severity in a subsequent prediction model.

Our models are dependent to a large extent on the practices at our institution and case-mix of our patient population. Since the validation was performed using the same population as the development data set, the prediction may be overestimated; nonetheless, development of a real-time alerting system from an internally validated model is still useful since the goal is to predict risk within a defined hospitalized population. Validation using an external population would be needed before applying this model for prediction in other hospital populations.

In this study, it was not possible to extract information from the medical history based on limitations in our previous EMR, and we relied exclusively on the hospital problem list, which may not be a complete reflection of the patient’s clinical conditions due to undercoding. Including all laboratory results regarding renal function, rather than simply using admission-day values, would improve the classification of AKI and CKD, and could account for the impact of hospital-acquired AKI on incident risk of hypoglycemia. We did not have information about administered medications containing dextrose, a potentially important confounder. We chose to exclude patients who received concurrent intravenous and subcutaneous insulin on the index day since patients on intravenous insulin infusions are more likely to be identified as at risk of hypoglycemia according to a protocol requiring hourly BG checks; however, it is possible that such patients might not differ significantly from the population included in our models and could therefore still benefit from a real-time informatics alert. We are currently working on a prediction model using data from our present EMR, which will include information about both subcutaneous and intravenous insulin. Finally, it is possible that there are other unknown predictors or confounders of hypoglycemia that we have not accounted for in our models. Extracting a larger amount of information from the EMR, including all laboratory results and medications, could reveal unexpected associations with hypoglycemia risk. Our current EMR has a more robust data storage system that allows clinicians to aggregate information about clinical conditions from multiple sources. Given the complexity of these models with multiple variables that require mathematical processing, they could only be practically applied in clinical practice with the concurrent use of an automated electronic calculator.

There are several strengths to this study. We adhered to consensus guidelines for development and validation of our models. Given the large sample size, we had sufficiently high event per predictor ratios in both models to minimize model overfitting.^34^ By restricting to patients on subcutaneous insulin in the non-critical care setting, our findings are generalizable to the majority of hospitalized patients with diabetes/hyperglycemia. We had very few missing data elements, but realize that our assumptions could have resulted in misclassification of some clinical conditions.

In conclusion, insulin-associated hypoglycemia in non-critically ill hospitalized adults can be predicted on the basis of EMR data. Further studies using more comprehensive information from these sources will likely improve the predictive accuracy of these models. Integration of such models into the EMR could increase the safety of hospitalized insulin-treated patients by alerting providers in real time about these high-risk patients.
