# Supplementary material for: Development and validation of a prediction model for insulin-associated hypoglycemia in non-critically ill hospitalized adults
Source: BMJ Open Diabetes Res Care. 2018 Mar 2;6(1):e000499. doi: 10.1136/bmjdrc-2017-000499 (PMC5841507; doi:10.1136/bmjdrc-2017-000499)
Supplement: Supplementary file 1 [file bmjdrc-2017-000499supp001.docx]

**Supplementary Materials**

*Model Building Strategy*

First, we used simple logistic regression analysis to explore the unadjusted association between outcomes and predictors in the development dataset (Supplemental Table S2). Collinearity between continuous measures was assessed by linear regression, with r >0.95 indicating collinearity.

For continuous predictors, a Lowess smoother function was used to assess linearity of predictors with probability of outcomes. Piecewise linear regression with spline terms was used for variables that did not appear to have a linear relationship with the probability of the outcomes by visual inspection of the Lowess smoother curve.

For a continuous variable *x* with one knot (*k*), we generated a new variable such that the linear segments represent the function for values of *x* within the two linear segments. New variables (k1 and k2) were created for each segment of the continuous variable as follows:

k1=$\left\{ \begin{aligned} x if x \leq k1 \\ k1 if x>k1 \end{aligned} \right.$ k2= $\left\{ \begin{aligned} \left( x-k1 \right) if x>k1 \\ 0 if x \leq k1 \end{aligned} \right.$

For a continuous variable *x* with 2 or more knots (k), new variables were created for the k+n linear segments as follows:

k1=$\left\{ \begin{aligned} x if x \leq k1 \\ k1 if x>k1 \end{aligned} \right.$ k2= $\left\{ \begin{aligned} 0 if x \leq k1 \\ (x-k1) if k1<x<k2 \\ (k2-k1) if x \geq k2 \end{aligned} \right.$ … kn=$\left\{ \begin{aligned} 0 if x\leq k2 \\ (kn-k2) if x>kn \end{aligned} \right.$

Continuous measures were scaled so that unit changes would be clinically interpretable in logistic regression models: age per 10 years, insulin per 0.1 units/kg, mean BG per 10 mg/dl, and CV_BG_ per 10% increments.

Backwards stepwise selection was used for selection of predictor variables in a multivariable logistic regression model, with a significance level of 0.2 for addition of variables and 0.1 for removal. Continuous measures with spline terms were specified as grouped variables such that they were all either included or removed from the model. When fitting the model, continuous measures were evaluated with and without spline terms.

The Akaike information criterion (AIC), which places less emphasis on parsimony than the Bayesian information criterion (BIC), was used to compare the candidate models[23]. As a guiding principle, greater emphasis was placed on model accuracy than parsimony, since we used predictors that could be automatically extracted from the EMR and calculated for clinicians. In addition, we considered the event per variable (EPV) ratio (i.e. number of hypoglycemic events per number of variables in the model) when selecting predictors and aimed for an EPV ratio of 10 or more to avoid model overfitting.


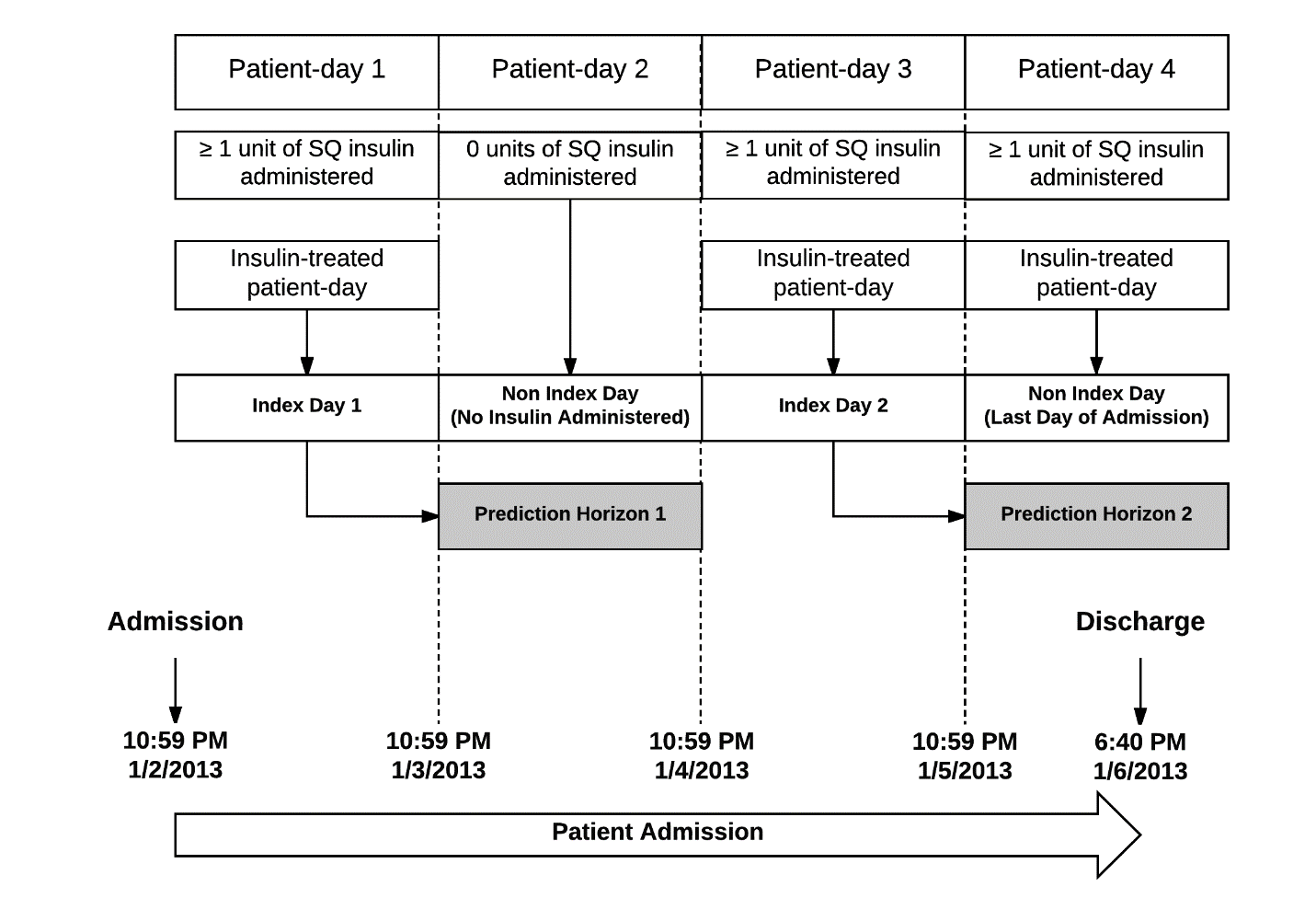


# Supplemental Figure S1. Prediction Horizon.

Illustration of patient-day, insulin-treated day, index day, and prediction horizon definitions used in prediction models. Patient-days defined as 24 hour increments from the admission date/time. Insulin-treated patient-days defined as any days in which at least 1 unit of SQ insulin was administered. Index days defined as insulin-treated patient-days in which there was no concurrent administration of intravenous insulin, total parenteral nutrition, or insulin pump use and which were followed by a patient-day (i.e. not occurring on the last day of admission). Prediction horizon defined as 24-hour window after index day.

# **Supplemental Table S1.** Definitions and data sources for outcomes and predictors.

| **Variable** | **Data Type** | **Definition** | **EMR Data Source (POE Sunrise)** | **When Collected or Assessed** |
| --- | --- | --- | --- | --- |
| Biochemical Hypoglycemia | Binary: 1= Yes; 0= No | Any BG ≤70 mg/dl in prediction horizon | POC or serum BG (Laboratory Results) | Within 24 hours after index day |
| Clinically significant hypoglycemia | Binary: 1= Yes; 0= No | Any BG <54 mg/dl in prediction horizon | POC or serum BG (Laboratory Results) | Within 24 hours after index day |
| Age | Continuous | Age in years at time of admission | Demographics/Visit Data | Patient-day 1 |
| Sex | Binary: 1= Male; 0= Female | Male/ Female | Demographics/Visit Data | Patient-day 1 |
| Race | Categorical:  0= White  1= Black  2= Asian  3= Other | White, Black/African American, Asian, or Other (All Other Races, American Indian/Alaskan Native, Hawaiin/Pacific Islander, Multi-racial, Unknown, Declined to Answer) | Demographics/Visit Data | Patient-day 1 |
| Weight, kg | Continuous | Weight | Height/Weight | Patient-day 1 |
| Basal insulin, units | Continuous | Sum of basal, intermediate-acting, and 70% of pre-mixed insulins doses on index day, expressed in units | Medication Administration Record (MAR) | Index day |
| Nutritional insulin, units | Continuous | Sum of nutritional aspart and 30% of total pre-mixed insulins administered on index day, expressed in units | Medication Administration Record (MAR) | Index day |
| Correctional insulin, units | Continuous | Sum of administered correctional aspart on index day, expressed in units | Medication Administration Record (MAR) | Index day |
| Total daily dose, units | Continuous | Sum of administered basal, nutritional, and correctional doses on index day, expressed in units | Medication Administration Record (MAR) | Index day |
| High dose SSI | Binary: 1= Yes; 0= No | High dose correctional insulin scale ordered at any time during index day | Medication Orders | Index day |
| Admitting Service (Medical vs. Surgical) | Binary: 0= Medical Service; 1= Surgical Service | Admitted to a non-critical care medical service: internal/general medicine, psychiatry (affective disorders, eating disorders, motivated behaviors, schizophrenia), cardiology, gastroenterology, geriatrics, hematological oncology, hematology, hospitalist service, infectious diseases, interventional radiology, medical oncology, neurology, pain treatment, palliative care, pulmonary, rehabilitation, nephrology)  Admitted to a non-critical care surgical service: orthopedics, cardiac surgery, dental surgery, GI surgery, neurosurgery, ophthalmology, head and neck surgery, plastic surgery, general surgery, surgical oncology, endocrine surgery, thoracic surgery, transplant surgery, urology, vascular surgery | Visit Data: Service | Patient-day 1 |
| Type 1 diabetes/pancreatectomy | Binary: 1= Yes; 0= No | ICD-9 codes*:  Type 1 diabetes:  250.01, 250.41, 250.43, 250.51, 250.53, 250.61, 250.63, 250.71, 250.73, 250.91, 250.93  Postsurgical hypoinsulinemia: 251.3 | Client Visit 3 (CV3) Health Issue Declaration Table Diagnostic Code | Status marked active on or prior to Patient-day 1 |
| Type 2 diabetes | Binary: 1= Yes; 0= No | ICD-9 code 250, 250.02, 250.10, 250.12, 250.20, 250.22, 250.30, 250.32, 250.40, 250.42, 250.50, 250.52, 250.60, 250.62, 250.70, 250.72, 250.80, 250.82, 250.90, 250.92 | Client Visit 3 (CV3) Health Issue Declaration Table Diagnostic Code | Status marked active on or prior to Patient-day 1 |
| Digestive disease | Binary: 1= Yes; 0= No | ICD-9 codes:  Symptoms involving digestive system: 787.X Symptoms involving abdomen and pelvis: 789.X  Intestinal obstruction: 560.X | Client Visit 3 (CV3) Health Issue Declaration Table Diagnostic Code | Patient-day 1 (admission diagnosis) |
| Liver disease | Binary: 1= Yes; 0= No | ICD-9 codes:  Alcohol dependence syndrome: 303.X  Acute and subacute necrosis of liver: 570.X  Chronic liver disease and cirrhosis: 571.X  Other disorders of liver: 573 | Client Visit 3 (CV3) Health Issue Declaration Table Diagnostic Code | Patient-day 1 (admission diagnosis) |
| Congestive heart failure | Binary: 1= Yes; 0= No | ICD-9 code: 428.X | Client Visit 3 (CV3) Health Issue Declaration Table Diagnostic Code | Patient-day 1 (admission diagnosis) |
| Pancreatic disease | Binary: 1= Yes, 0=No | ICD-9 code codes:  Diseases of pancreas: 577.X  Malignant neoplasm of pancreas: 157.9 | Client Visit 3 (CV3) Health Issue Declaration Table Diagnostic Code | Patient-day 1 (admission diagnosis) |
| Acute kidney injury | Binary: 1= Yes; 0=No | ICD-9 code 584.X | Client Visit 3 (CV3) Health Issue Declaration Table Diagnostic Code | Patient-day 1 (admission diagnosis) |
| Chronic kidney disease (CKD) | Categorical: |  |  |  |
| None | 0= None | GFR ≥60 ml/min/1.73 m^2^ | Laboratory Results | Patient-day 1 |
| Stage 3 | 1= Stage 3 | GFR 30-59 ml/min/1.73 m^2^ | Laboratory Results | Patient-day 1 |
| Stage 4 | 2= Stage 4 | GFR 15-29 ml/min/1.73 m^2^ | Laboratory Results | Patient-day 1 |
| Stage 5 | 3= Stage 5 | GFR <15 15-29 ml/min/1.73 m^2^ | Laboratory Results | Patient-day 1 |
| Mean BG, mg/dl | Continuous | Average of all POC and serum BG readings on index day | Laboratory Results | Index day |
| CV of BG, % | Continuous | (Standard deviation of all POC and serum BGs on patient-day / Mean BG on index day)*100 | Laboratory Results | Index day |
| Nadir BG, mg/dl | Continuous | Lowest POC or serum BG on index day | Laboratory Results | Index day |
| Admission Mean BG, mg/dl | Continuous | Average of all POC and serum BG readings on all patient days prior to and including index day | Laboratory Results | All patient days prior to and including index day |
| Admission CV of BG, % | Continuous | Standard deviation of all POC and serum BGs on all patient days prior to and including index day / Admission Mean BG)*100 | Laboratory Results | All patient days prior to and including index day |
| Admission Nadir BG, mgd/l | Continuous | Lowest POC or serum BG on all patient days prior to and including index day | Laboratory Results | All patient days prior to and including index day |
| Diet order | Categorical: |  |  |  |
| NPO | 0= NPO | 0= NPO: Nil per os (NPO) diet ordered at any time during index day | Nutrition order management | Index Day |
| Carbohydrate controlled | 1= Carbohydrate controlled | 1=Carbohydrate controlled diet ordered without any bolus tube feed or NPO order during index day | Nutrition order management | Index Day |
| Regular/other | 2= Regular/other | 2= Regular or other diet ordered without any bolus tube feed, carbohydrate controlled diet, or bolus tube feed ordered during index day | Nutrition order management | Index Day |
| Bolus tube feed | 3= Bolus tube feed | 3= Bolus tube feed ordered during index day without any diet order for NPO during index day | Nutrition order management | Index Day |
| Steroid use | Binary: 1= Yes; 0=No | Administration of IV, IM, or oral dexamethasone, prednisone, prednisolone, or methylprednisolone on index day | Medication Administration Record (MAR) | Index Day |
| BG= blood glucose; POC= point-of-care; kg= kilogram; SSI= sliding scale insulin; GFR= glomerular filtration rate; CV= coefficient of variation; NPO= nil per os; IV= intravenous; IM= intramuscular. ICD= International Classification of Diseases.  *ICD-10 was released on October 1, 2015 and study end date was December 31, 2015. For records after October 1, 2015, the ICD-9 code corresponding to the ICD-10 code was extracted from the CV3 Health Declaration Table, as both codes were available in this data source. | | | | |

# **Supplemental Table S2**. Univariate (unadjusted) associations of candidate predictors and hypoglycemic outcomes in development dataset.

|  |  |  | **Model 1: BG ≤ 70 mg/dl** | | **Model 2: BG <54 mg/dl** | |
| --- | --- | --- | --- | --- | --- | --- |
|  | Unit of change | Spline Knot | OR (95% CI) | P-value | OR (95% CI) | P-value |
| Age | 10 years |  |  |  |  |  |
| age_1_ |  | ≤ 40 | **0.71 (0.65-0.78)** | <0.001 | **0.64 (0.55-0.75)** | <0.001 |
| age_2_ |  | >40 | **0.87 (0.85-0.90)** | <0.001 | **0.85 (0.81-0.90)** | <0.001 |
| Female |  |  | **1.24 (1.16-1.32)** | <0.001 | 1.12 (1.00-1.26) | 0.060 |
| Race |  |  |  |  |  |  |
| White |  |  | 1.00 (ref) | - | 1.00 (ref) |  |
| Black |  |  | **1.39 (1.30-1.49)** | <0.001 | **1.42 (1.25-1.61)** | <0.001 |
| Asian |  |  | **0.78 (0.61-0.99)** | 0.046 | **0.58 (0.34-0.99)** | 0.045 |
| Other |  |  | **1.27 (1.13-1.42)** | <0.001 | **1.35 (1.10-1.66)** | 0.005 |
| Weight | 10 kg |  |  |  |  |  |
| weight_1_ |  | ≤ 80 | **0.78 (0.76-0.80)** | <0.001 | **0.74 (0.70-0.78)** | <0.001 |
| weight_2_ |  | >80 | **0.94 (0.92-0.96)** | <0.001 | **0.87 (0.82-0.91)** | <0.001 |
| Admission to surgical service |  |  | **0.65 (0.61-0.69)** | <0.001 | **0.58 (0.51-0.66)** | <0.001 |
| Basal insulin dose | 0.1 units/kg |  |  |  |  |  |
| basal_1_ |  | ≤ 0.2 | **2.45 (2.34-2.56)** | <0.001 | **2.96 (2.71-3.25)** | <0.001 |
| basal_2_ |  | ≤ 0.8 | **1.11 (1.07-1.14)** | <0.001 | **1.11 (1.06-1.16)** | <0.001 |
| basal_3_ |  | ≤ 1.3 | 0.95 (0.85-1.05) | 0.295 | 0.94 (0.80-1.12) | 0.503 |
| basal_4_ |  | ≤ 1.6 | 1.27 (0.93-1.72) | 0.133 | **1.76 (1.12-2.78)** | 0.015 |
| basal_5_ |  | >1.6 | 0.91 (0.71-1.17) | 0.468 | 0.41 (0.12-1.39) | 0.152 |
| Nutritional insulin dose | 0.1 units/kg |  |  |  |  |  |
| nutritional_1_ |  | ≤ 0.6 | **1.35 (1.32-1.38)** | <0.001 | **1.39 (1.34-1.44)** | <0.001 |
| nutritional_2_ |  | ≤ 0.9 | 0.90 (0.77-1.04) | 0.148 | 0.87 (0.68-1.12) | 0.287 |
| nutritional_3_ |  | ≤ 1.1 | 0.65 (0.41-1.01) | 0.057 | 0.55 (0.24-1.26) | 0.159 |
| nutritional_4_ |  | >1.1 | 1.15 (0.93-1.53) | 0.206 | 1.33 (0.98-1.80) | 0.067 |
| Correctional insulin dose | 0.1 units/kg |  |  |  |  |  |
| correctional_1_ |  | ≤ 0.04 | **0.12 (0.10-0.17)** | <0.001 | **0.33 (0.19-0.55)** | <0.001 |
| correctional_2_ |  | ≤ 0.7 | **1.25 (1.21-1.30)** | <0.001 | **1.38 (1.31-1.45)** | <0.001 |
| correctional_3_ |  | ≤ 0.9 | 0.59 (0.33-1.07) | 0.082 | 0.88 (0.40-1.91) | 0.738 |
| correctional_4_ |  | >0.9 | 1.08 (0.73-1.60) | 0.687 | 0.22 (0.01-7.85) | 0.410 |
| High dose SSI |  |  | **2.78 (2.57-3.01)** | <0.001 | **3.79 (3.21-4.47)** | <0.001 |
| Index day mean BG | 10 mg/dl |  |  |  |  |  |
| mean_1_ |  | ≤ 100 | **0.44 (0.39-0.50)** | <0.001 | **0.47 (0.39-0.56)** | <0.001 |
| mean_2_ |  | ≤ 150 | **0.77 (0.75-0.79)** | <0.001 | **0.81 (0.78-0.85)** | <0.001 |
| mean_3_ |  | >150 | **1.01 (1.01-1.03)** | <0.001 | **1.05 (1.04-1.07)** | <0.001 |
| Index day nadir BG | 10 mg/dl |  |  |  |  |  |
| nadir_1_ |  | ≤ 88 | **0.70 (0.68-0.72)** | <0.001 | **0.63 (0.60-0.66)** | <0.001 |
| nadir_2_ |  | ≤ 100 | **0.37 (0.34-0.40)** | <0.001 | **0.45 (0.38-0.53)** | <0.001 |
| nadir_3_ |  | >100 | **0.95 (0.93-0.96)** | <0.001 | 0.99 (0.96-1.01) | 0.307 |
| Index day CV of BG | 10% |  |  |  |  |  |
| cv_1_ |  | ≤ 10 | 0.69 (0.46-1.04) | 0.079 | 0.73 (0.31-1.75) | 0.484 |
| cv_2_ |  | ≤ 20 | **3.47 (2.99-4.02)** | <0.001 | **3.51 (2.61-4.73)** | <0.001 |
| cv_3_ |  | >20 | **1.58 (1.54-1.62)** | <0.001 | **1.69 (1.62-1.76)** | <0.001 |
| Admission mean BG | 10 mg/dl |  |  |  |  |  |
| admission mean_1_ |  |  | **0.52 (0.38-0.71)** | <0.001 | **0.54 (0.35-0.83)** | 0.005 |
| admission mean_2_ |  |  | **0.67 (0.62-0.72)** | <0.001 | **0.82 (0.69-0.97)** | 0.022 |
| admission mean_3_ |  |  | **1.04 (1.04-1.05)** | <0.001 | **1.08 (1.06-1.09)** | <0.001 |
| Admission nadir BG | 10 mg/dl |  |  |  |  |  |
| admission nadir_1_ |  | ≤ 100 | **0.69 (0.68-0.70)** | <0.001 | **0.65 (0.64-0.67)** | <0.001 |
| admission nadir_2_ |  | ≤ 400 | **0.98 (0.96-0.99)** | 0.016 | **1.03 (1.00-1.07)** | 0.033 |
| admission nadir_3_ |  | >400 | 1.01 (0.80-1.27) | 0.960 | 1.00 (0.78-1.28) | 0.975 |
| Admission CV of BG | 10% |  |  |  |  |  |
| admission cv_1_ |  | ≤ 18 | **1.45 (1.16-1.82)** | 0.001 | 1.31 (0.84-2.05) | 0.230 |
| admission cv_2_ |  | >18 | **1.98 (1.92-2.03)** | <0.001 | **2.08 (1.99-2.17)** | <0.001 |
| Diet orders |  |  |  |  |  |  |
| NPO |  |  | 1.00 (ref) | - | 1.00 (ref) | - |
| Carb controlled |  |  | **1.18 (1.05-1.33)** | 0.007 | 1.11 (0.89-1.39) | 0.344 |
| Regular or other |  |  | **1.09 (1.01-1.19)** | 0.026 | 0.97 (0.84-1.12) | 0.697 |
| Bolus tube feeds |  |  | **0.79 (0.63-0.99)** | 0.041 | 0.90 (0.61-1.32) | 0.575 |
| Type 1 diabetes / pancreatectomy |  |  | **4.19 (3.86-4.56)** | <0.001 | **7.19 (6.30-8.20)** | <0.001 |
| Type 2 diabetes |  |  | **1.95 (1.80-2.12)** | <0.001 | **2.10 (1.78-2.47)** | <0.001 |
| AKI |  |  | **1.88 (1.50-2.35)** | <0.001 | **1.98 (1.33-2.96)** | 0.001 |
| CKD |  |  |  |  |  |  |
| None |  |  | **1.00 (ref)** | - | 1.00 (ref) | - |
| Stage 3 |  |  | **1.39 (1.28-1.51)** | <0.001 | **1.39 (1.20-1.62)** | <0.001 |
| Stage 4 |  |  | **1.92 (1.74-2.11)** | <0.001 | **2.13 (1.79-2.53)** | <0.001 |
| Stage 5 |  |  | **2.12 (1.91-2.37)** | <0.001 | **2.55 (2.11-3.08)** | <0.001 |
| Liver disease |  |  | **0.48 (0.33-0.68)** | <0.001 | 0.57 (0.31-1.07) | 0.081 |
| CHF |  |  | 0.97 (0.81-1.16) | 0.711 | 0.77 (0.53-1.12) | 0.173 |
| Digestive disease |  |  | **1.67 (1.47-1.90)** | <0.001 | **1.88 (1.51-2.36)** | <0.001 |
| Pancreatic disease |  |  | **0.75 (0.59-0.96)** | 0.020 | **0.55 (0.32-0.93)** | 0.025 |
| Steroids on index day |  |  | 1.02 (0.96-1.09) | 0.546 | **0.87 (0.77-0.98)** | 0.027 |
| BG= blood glucose; OR= odds ratio; CI= confidence interval ; ref= reference; kg= kilogram; SSI= sliding scale insulin; CV= coefficient of variation; NPO= nil per os; AKI= acute kidney injury; CKD= chronic kidney disease; CHF= congestive heart failure. Bolded values signify P<0.05. | | | | | | |

# Supplemental Table S3. Example calculation of individual patient risk (mock case) using Model 1.

| **Variables on index day:**   - 35 year-old black male admitted to hospitalist (medical) service for CHF exacerbation - Weight 74.2 kg - Admission diagnoses/problem list:   - Acute kidney injury   - Type 1 diabetes - SQ insulin doses:   - Basal dose= 25 units= 25 units/74.2 kg = 0.34 units/kg   - Nutritional dose= 0 units = 0 units/74.2 kg = 0.00 units/kg   - Correctional dose= 6 units = 6 units/74.2 kg= 0.08 units/kg - High dose SSI - Mean BG= 127 mg/dl - Nadir BG= 75 mg/dl - CV_BG_= 37.1 % - NPO diet - Treated with steroids   **Variables on admission (up to and including index day):**   - Admission Nadir BG= 62 mg/dl - Admission CV_BG_= 28.5% | | | | |
| --- | --- | --- | --- | --- |
| **Logistic Odds Equation for Model 1 (BG ≤70 mg/dl):**  Log odds (BG $\leq70\frac{mg}{dl})=$2.140 + 0.066* age_1_ + -0.045*age_2_ + -0.136*weight_1_ + -0.079*weight_2_ + -0.091*service + 0.617*basal_1_ + 0.130*basal_2_ + -0.050*basal_3_ + 0.249*basal_4_ + 0.755*basal_5_ + 0.048*nutritional_1_ + 0.048*nutritional_2_ + -0.534*nutritional_3_ + 0.071*nutritional_4_ + -0.365*correctional_1_+ 0.041*correctional_2_ + -0.336*correctional_3_ + 0.159*correctional_4_ + 0.135*high dose SSI +-0.312* mean_1_ + -0.155*mean_2_ + -0.011*mean_3_ + -0.011*nadir_1_+ -0.440*nadir_2_ + -0.070*nadir_3_ + -0.077*cv_1_ + 0.541*cv_2_ + 0.006*cv_3_ + -0.134*admission nadir_1_ +-0.029*admission nadir_2_ + -0.180*admission nadir_3_ + -0.249* admission cv_1_ + 0.171*admission cv_2_ + -0.218*carb controlled + -0.235*regular diet + -0.382*bolus tube +0.357*T1DM + 0.224* T2DM + 0.238*AKI + 0.224*Stage 3 CKD + 0.418*Stage 4 CKD + 0.565* Stage 5 CKD + -0.363*Liver disease + 0.184*Digestive disease.  Estimated Probability of BG $\leq70\frac{mg}{dl})$can be calculated from coefficients (b) as follows:  $P\left( BG\leq70\frac{mg}{dl} \right)=\frac{e^{(b0+b1x1+b2x2+\ldots)}}{1+ e^{(b0+b1x1+b2x2+\ldots)}}$  **Decision Rule: Classified at-risk of outcome if estimated probability ≥ 0.038** | | | | |
| **Variable (X)** | **Definition of X** | **Patient Value for X** | **Scaled Value for X** | **Term in Log Odds Equation** |
| Intercept | - | - | - | **2.140** |
| age_1_ | age if age ≤40; 40 if age >40 | 35 | 35/10= 3.5 | 0.066 * 3.5= **0.264** |
| age_2_ | (age - 40) if age >40; 0 if age ≤40 | 0 | 0/10=0 | -0.045*0= **0** |
| weight_1_ | weight if weight ≤80; 80 if weight >80 | 74.2 | 74.2/10= 7.42 | -0.136*7.42= **-1.00912** |
| weight_2_ | (weight – 80) if weight >80; 0 if weight ≤80 | 0 | 0/10= 0 | -0.079*0= **0** |
| service | 1= surgical service; 0= medical service | 0 | - | -0.091*0 = **0** |
| basal_1_ | basal dose (units/kg) if basal dose ≤0.2;  0.2 if basal dose >0.2 | 0.2 | 0.2*10 = 2 | 0.617* 2= **1.234** |
| basal_2_ | 0 if basal dose (units/kg) ≤0.2;  (basal dose - 0.2) if 0.2 < basal dose ≤0.8;  0.6 if basal dose >0.8 | 0.34 – 0.20 = 0.14 | 0.14 * 10= 1.4 | 0.130 * 1.4= **0.182** |
| basal_3_ | 0 if basal dose (units/kg) ≤0.8;  (basal dose - 0.8) if 0.8 < basal dose ≤1.3;  0.5 if basal dose >1.3 | 0 | 0 * 10= 0 | 0.050 * 0 = **0** |
| basal_4_ | 0 if basal dose (units/kg) ≤1.3;  (basal dose – 1.3) if 1.3< basal dose ≤1.6;  0.3 if basal dose >1.6 | 0 | 0 * 10=0 | 0.249* 0= **0** |
| basal_5_ | 0 if basal dose (units/kg) ≤1.6;  (basal dose – 1.6) if basal dose >1.6 | 0 | 0 * 10= 0 | 0.755 * 0 =**0** |
| nutritional_1_ | nutritional dose (units/kg) if nutritional dose ≤0.6;  0.6 if nutritional dose >0.6 | 0 | 0 * 10= 0 | 0.048* 0 =**0** |
| nutritional_2_ | 0 if nutritional dose (units/kg) ≤0.6;  (nutritional dose - 0.6) if 0.6 < nutritional dose ≤0.9;  0.3 if nutritional dose >0.9 | 0 | 0 * 10= 0 | 0.048 * 0 = **0** |
| nutritional_3_ | 0 if nutritional dose (units/kg) ≤0.9;  (nutritional dose - 0.9) if 0.9 < nutritional dose ≤1.1;  0.2 if nutritional dose >1.1 | 0 | 0 * 10= 0 | -0.534 * 0 =**0** |
| nutritional_4_ | 0 if nutritional dose (units/kg) ≤1.1;  (nutritional dose – 1.1) if nutritional dose >1.1 | 0 | 0 * 10= 0 | 0.071* 0 = **0** |
| correctional_1_ | correctional dose (units/kg) if correctional dose ≤0.04;  0.04 if correctional dose >0.04 | 0.04 | 0.04*10=0.4 | -0.365 * 0.4= **-0.146** |
| correctional_2_ | 0 if correctional dose (units/kg) ≤0.04;  (correctional dose - 0.04) if 0.04 < correctional dose ≤0.7;  0.66 if correctional dose >0.7 | 0.08-0.04= 0.04 | 0.04*10= 0.4 | 0.041*0.4= **0.0164** |
| correctional_3_ | 0 if correctional dose (units/kg) ≤0.7;  (correctional dose - 0.7) if 0.7 < correctional dose ≤0.9;  0.2 if correctional dose >0.9 | 0 | 0*10=0 | -0.336*0=**0** |
| correctional_4_ | 0 if correctional dose (units/kg) ≤0.9;  (correctional dose - 0.9) if correctional dose >0.9 | 0 | 0*10=0 | 0.159*0=**0** |
| high dose SSI | 1= if yes; 0 = no | 1 | - | 0.135*1= **0.135** |
| mean_1_ | mean BG if mean BG ≤100; 100 if mean BG >100 | 100 | 100/10=10 | -0.312*10= **-3.12** |
| mean_2_ | 0 if mean BG ≤100;  (mean BG – 100) if 100 < mean BG ≤150;  50 if mean BG >150 | 127-100= 27 | 27/10= 2.7 | -0.155*2.7= **-0.3888** |
| mean_3_ | 0 if mean BG ≤150;  (mean BG- 150) if mean BG >150 | 0 | 0/10=0 | -0.011*0= **0** |
| nadir_1_ | nadir BG if nadir BG ≤88; 88 if mean BG >88 | 75 | 75/10=7.5 | -0.011*7.5= **-0.0825** |
| nadir_2_ | 0 if nadir BG ≤100;  (mean BG – 88) if 88 < mean BG ≤100;  12 if mean BG >100 | 0 | 0/10=0 | -0.440*0 = **0** |
| nadir_3_ | 0 if nadir BG ≤100;  (mean BG – 100) if mean BG >100 | 0 | 0/10=0 | -0.070*0= **0=0** |
| cv_1_ | CV_BG_ if CV_BG_ ≤10;  10 if CV_BG_ >10 | 10 | 10/10=1 | -0.077*1= **-0.077** |
| cv_2_ | 0 if CV_BG_ ≤10;  (CV_BG_ – 10) if 10 < CV_BG_ ≤20;  10 if CV_BG_ >20 | 10 | 10/10=1 | 0.541*1= **0.541** |
| cv_3_ | 0 if CV_BG_ ≤20;  (CV_BG_ – 20) if CV_BG_ >20 | 37.1-20= 17.1 | 17.1/10=1.71 | 0.006*1.71= **0.01026** |
| admission nadir_1_ | admission nadir BG if admission nadir BG ≤100;  100 if admission nadir BG >100 | 62 | 62/10= 6.2 | -0.134*6.2= **-0.8308** |
| admission nadir_2_ | 0 if admission nadir BG ≤100;  (admission nadir BG – 100) if 100 < admission nadir BG ≤400;  300 if admission nadir BG >400 | 0 | 0/10= 0 | 0.029*0= **0** |
| admission nadir_3_ | 0 if admission nadir BG ≤400;  (admission nadir BG – 400) if admission nadir BG >400 | 0 | 0/10=0 | -0.180*0=**0** |
| admission cv_1_ | admission CV_BG_ if admission CV_BG_ ≤18;  18 if admission CV_BG_ >18 | 18 | 18/10=1.8 | -0.249*1.8=**-0.4482** |
| admission cv_2_ | 0 if admission CV_BG_ ≤18  (admission CV_BG_ – 18) if admission CV_BG_ >18 | 28.5-18=10.5 | 10.5/10=1.05 | 0.171*1.05= **0.17955** |
| carb controlled | 1= carb controlled diet; 0= NPO | 0 | - | -0.218*0=**0** |
| regular diet | 1= regular or other diet; 0 = NPO | 0 | - | -0.235*0= **0** |
| bolus tube | 1= bolus tube feed; 0 = NPO | 0 | - | -0.382*0= **0** |
| T1DM | 1= present; 0 = absent | 1 | - | 0.357*1= **0.357** |
| T2DM | 1= present; 0 = absent | 0 | - | 0.224*0= **0** |
| AKI | 1= present; 0 = absent | 1 | - | 0.238*1= **0.238** |
| Stage 3 CKD | 1= present; 0 = absent | 0 | - | 0.224* 0 =**0** |
| Stage 4 CKD | 1= present; 0 = absent | 0 | - | 0.418*0=**0** |
| Stage 5 CKD | 1= present; 0 = absent | 0 | - | 0.565*0=**0** |
| Liver disease | 1= present; 0 = absent | 0 | - | -0.363*0=**0** |
| Digestive disease | 1= present; 0 = absent | 0 | - | 0.184*0=**0** |
| Using terms above (bold values from last column), calculated probability is:  $P\left( BG\leq70\frac{mg}{dl} \right)=\frac{e^{(2.14+0.264+ -1.00912+1.234+0.182+ -0.146+0.0164+0.135+ -3.12+ -0.3888+ -0.077+0.541+0.01026+ -0.8308+ -0.4482+0.17955+0.357+0.238)}}{1+ e^{(2.14+0.264 + -1.00912+1.234+0.182+ -0.146+0.0164+0.135+ -3.12+ -0.3888+ -0.077+0.541+0.01026+ -0.8308+ -0.4482+0.17955+0.357+0.238)}}$  = $\frac{e^{-0.80571}}{1+ e^{-0.80571}}$ = **0.309**  **Since calculated probability of 0.309 is ≥ 0.038, patient would be classified as at risk for BG** $\boldsymbol{\leq70}\frac{\boldsymbol{mg}}{\boldsymbol{dl}}$ **within next 24 hours.** | | | | |
| Kg= kilogram; SSI= sliding scale insulin; SQ= subcutaneous; BG= blood glucose; CV= coefficient of variation; NPO= nil per os; T1DM= type 1 diabetes mellitus; T2DM= type 2 diabetes mellitus; AKI= acute kidney injury; CKD= chronic kidney disease | | | | |

# Supplemental Table S4. Example calculation of individual patient risk (mock case) using Model 2.

| **Variables on index day:**   - 65.4 year-old white female admitted to vascular surgery service for lower extremity arterial occlusion - Weight 86.2 kg - SQ insulin doses:   - Basal dose= 0 units= 0 units/ 76.2 kg = 0 units/kg   - Nutritional dose= 0 units = 0 units/76.2 kg = 0 units/kg   - Correctional dose= 7 units = 6 units/76.2 kg= 0.08 units/kg - Medium dose SSI - Mean BG= 227 mg/dl - Nadir BG= 192 mg/dl - CV_BG_= 13.7% - NPO diet   **Variables on admission (up to and including index day):**   - Admission Nadir BG= 126 mg/dl - Admission CV_BG_= 18.6% | | | | |
| --- | --- | --- | --- | --- |
| **Logistic Odds Equation for Model 2 (BG <54 mg/dl):**  Log odds (BG $<54 \frac{mg}{dl})=$0.580 + -0.280* female -0.160*weight_1_ + -0.151*weight_2_ + 0.697*basal_1_ + 0.141*basal_2_ + -0.046*basal_3_ + 0.609*basal_4_ + -1.238*basal_5_ + 0.049*nutritional_1_ + 0.035*nutritional_2_ + -0.674*nutritional_3_ + 0.176*nutritional_4_ + 0.182*high dose SSI +-0.220* mean_1_ + -0.122*mean_2_ + -0.030*mean_3_ + -0.099*nadir_1_+ -0.228*nadir_2_ + -0.076*nadir_3_ + 0.035*cv_1_ + 0.417*cv_2_ + 0.017*cv_3_ + -0.147*admission nadir_1_ + 0.035*admission nadir_2_ + -0.122*admission nadir_3_ + -0.311* admission cv_1_ + 0.159*admission cv_2_ + -0.406*carb controlled + -0.373*regular diet + -0.268*bolus tube +0.712*T1DM + 0.253* T2DM + 0.314*Stage 3 CKD + 0.588*Stage 4 CKD + 0.795* Stage 5 CKD + -0.170*steroids.  Estimated Probability of BG $<54 \frac{mg}{dl})$can be calculated from coefficients (b) as follows:  $P\left( BG<54 \frac{mg}{dl} \right)=\frac{e^{(b0+b1x1+b2x2+\ldots)}}{1+ e^{(b0+b1x1+b2x2+\ldots)}}$  **Decision Rule:** Classified at-risk of outcome if estimated probability ≥ 0.009 | | | | |
| **Variable (X)** | **Definition of X** | **Patient Value for X** | **Scaled Value for X** | **Term in Log Odds Equation** |
| Intercept | - | - | - | **0.580** |
| Female | 1= female; 0 = male | 1 | - | -0.280*1 = **-0.280** |
| weight_1_ | weight if weight ≤80; 80 if weight >80 | 80 | 80/10= 8.00 | -0.160*8.00= **-1.28** |
| weight_2_ | (weight – 80) if weight >80; 0 if weight ≤80 | 6.2 | 6.2/10= 0.62 | -0.151*0.62= **0.09** |
| basal_1_ | basal dose (units/kg) if basal dose ≤0.2;  0.2 if basal dose >0.2 | 0 | 0*10=0 | 0.697* 0= **0** |
| basal_2_ | 0 if basal dose (units/kg) ≤0.2;  (basal dose - 0.2) if 0.2 < basal dose ≤0.8;  0.6 if basal dose >0.8 | 0 | 0 * 10= 0 | 0.141* 0= **0** |
| basal_3_ | 0 if basal dose (units/kg) ≤0.8;  (basal dose - 0.8) if 0.8 < basal dose ≤1.3;  0.5 if basal dose >1.3 | 0 | 0 * 10= 0 | -0.046 * 0 = **0** |
| basal_4_ | 0 if basal dose (units/kg) ≤1.3;  (basal dose – 1.3) if 1.3< basal dose ≤1.6;  0.3 if basal dose >1.6 | 0 | 0 * 10=0 | 0.609* 0= **0** |
| basal_5_ | 0 if basal dose (units/kg) ≤1.6;  (basal dose – 1.6) if basal dose >1.6 | 0 | 0 * 10= 0 | -1.238 * 0 =**0** |
| nutritional_1_ | nutritional dose (units/kg) if nutritional dose ≤0.6;  0.6 if nutritional dose >0.6 | 0 | 0 * 10= 0 | 0.049* 0 =**0** |
| nutritional_2_ | 0 if nutritional dose (units/kg) ≤0.6;  (nutritional dose - 0.6) if 0.6 < nutritional dose ≤0.9;  0.3 if nutritional dose >0.9 | 0 | 0 * 10= 0 | 0.035 * 0 = **0** |
| nutritional_3_ | 0 if nutritional dose (units/kg) ≤0.9;  (nutritional dose - 0.9) if 0.9 < nutritional dose ≤1.1;  0.2 if nutritional dose >1.1 | 0 | 0 * 10= 0 | -0.674 * 0 =**0** |
| nutritional_4_ | 0 if nutritional dose (units/kg) ≤1.1;  (nutritional dose – 1.1) if nutritional dose >1.1 | 0 | 0 * 10= 0 | 0.176* 0 = **0** |
| high dose SSI | 1= if yes; 0 = no | 0 | - | 0.169*0= **0** |
| mean_1_ | mean BG if mean BG ≤100; 100 if mean BG >100 | 100 | 100/10=10 | -0.220*10= **-2.2** |
| mean_2_ | 0 if mean BG ≤100;  (mean BG – 100) if 100 < mean BG ≤150;  50 if mean BG >150 | 50 | 50/10= 5 | -0.122*5= **-0.61** |
| mean_3_ | 0 if mean BG ≤150;  (mean BG- 150) if mean BG >150 | 227-150= 77 | 77/10=7.7 | 0.030*7.7= **0.231** |
| nadir_1_ | nadir BG if nadir BG ≤88; 88 if mean BG >88 | 88 | 88/10=8.8 | -0.099*8.8= **-0.8712** |
| nadir_2_ | 0 if nadir BG ≤100;  (mean BG – 88) if 88 < mean BG ≤100;  12 if mean BG >100 | 12 | 12/10=1.2 | -0.228*1.2 = **-0.2736** |
| nadir_3_ | 0 if nadir BG ≤100;  (mean BG – 100) if mean BG >100 | 192-100= 92 | 92/10=9.2 | -0.076*9.2=**-0.6992** |
| cv_1_ | CV_BG_ if CV_BG_ ≤10;  10 if CV_BG_ >10 | 10 | 10/10=1 | 0.035*1= **0.035** |
| cv_2_ | 0 if CV_BG_ ≤10;  (CV_BG_ – 10) if 10 < CV_BG_ ≤20;  10 if CV_BG_ >20 | 13.7-10= 3.7 | 3.7/10= 0.37 | 0.417* 0.37= **0.15429** |
| cv_3_ | 0 if CV_BG_ ≤20;  (CV_BG_ – 20) if CV_BG_ >20 | 0 | 0/10= 0 | 0.017*0= **0** |
| admission nadir_1_ | admission nadir BG if admission nadir BG ≤100;  100 if admission nadir BG >100 | 100 | 100/10= 10 | -0.147*10= **-1.47** |
| admission nadir_2_ | 0 if admission nadir BG ≤100;  (admission nadir BG – 100) if 100 < admission nadir BG ≤400;  300 if admission nadir BG >400 | 126-100= 26 | 26/10= 2.6 | 0.035*2.6= **0.091** |
| admission nadir_3_ | 0 if admission nadir BG ≤400;  (admission nadir BG – 400) if admission nadir BG >400 | 0 | 0/10=0 | -0.122*0=**0** |
| admission cv_1_ | admission CV_BG_ if admission CV_BG_ ≤18;  18 if admission CV_BG_ >18 | 18 | 18/10=1.8 | -0.311*1.8= **-0.5598** |
| admission cv_2_ | 0 if admission CV_BG_ ≤18  (admission CV_BG_ – 18) if admission CV_BG_ >18 | 18.6-18=0.6 | 0.6/10=0.06 | 0.159*0.06= **0.00954** |
| carb controlled | 1= carb controlled diet; 0= NPO | 0 | - | -0.406*0=**0** |
| regular diet | 1= regular or other diet; 0 = NPO | 0 | - | -0.373*0= **0** |
| bolus tube | 1= bolus tube feed; 0 = NPO | 0 | - | -0.268*0= **0** |
| T1DM | 1= present; 0 = absent | 0 | - | 0.712*0= **0** |
| T2DM | 1= present; 0 = absent | 0 | - | 0.253*0= **0** |
| Stage 3 CKD | 1= present; 0 = absent | 0 | - | 0.314* 0 =**0** |
| Stage 4 CKD | 1= present; 0 = absent | 0 | - | 0.588*0=**0** |
| Stage 5 CKD | 1= present; 0 = absent | 0 | - | 0.795*0=**0** |
| Steroids | 1= steroid use on index day; 0= no steroid use on index day | 0 | - | -0.170*0=**0** |
| Using terms above (bold values from last column), calculated probability is:  $P\left( BG<54\frac{mg}{dl} \right)=\frac{e^{(0.580+ -0.28+ -1.28+0.09 + -2.2 + -0.61+0.231+ -0.8712+ -0.2736+ -0.6992+0.035+0.15429 + -1.47+0.091+ -0.5598+0.00954 )}}{1+ e^{(0.580+ -0.28+ -1.28+0.09 + -2.2 + -0.61+0.231+ -0.8712+ -0.2736+ -0.6992+0.035+0.15429 + -1.47+0.091+ -0.5598+0.00954)}}$  = $\frac{e^{-5.6827}}{1+ e^{-5.6827}}$ = **0.0009**  **Since calculated probability of 0.0009 is ≤ 0.009, patient would be classified as not at risk for BG** $\boldsymbol{<54}\frac{\boldsymbol{mg}}{\boldsymbol{dl}}$ **within next 24 hours.** | | | | |
| Kg= kilogram; SSI= sliding scale insulin; SQ= subcutaneous; BG= blood glucose; CV= coefficient of variation; NPO= nil per os; T1DM= type 1 diabetes mellitus; T2DM= type 2 diabetes mellitus; AKI= acute kidney injury; CKD= chronic kidney disease | | | | |
